# Activation of Human Osteoblasts via Different Bovine Bone Substitute Materials With and Without Injectable Platelet Rich Fibrin *in vitro*

**DOI:** 10.3389/fbioe.2021.599224

**Published:** 2021-02-17

**Authors:** Solomiya Kyyak, Sebastian Blatt, Eik Schiegnitz, Diana Heimes, Henning Staedt, Daniel G. E. Thiem, Keyvan Sagheb, Bilal Al-Nawas, Peer W. Kämmerer

**Affiliations:** ^1^Department of Oral- and Maxillofacial Surgery, University Medical Center Mainz, Mainz, Germany; ^2^Private Practice, University Medical Center Rostock, Rostock, Germany; ^3^Department of Prosthodontics and Materials Science, University Medical Center Rostock, Rostock, Germany

**Keywords:** bone substitute, bovine bone, platelet rich fibrin (PRF), *in vitro*, osteoblast, vitality, proliferation, PCR

## Abstract

**Introduction:**

The aim of the *in vitro* study was to compare the effect of four bovine bone substitute materials (XBSM) with and without injectable platelet-reach fibrin for viability and metabolic activity of human osteoblasts (HOB) as well as expression of alkaline phosphatase (ALP), bone morphogenetic protein 2 (BMP-2), and osteonectin (OCN).

**Materials and Methods:**

Cerabone^®^ (CB), Bio-Oss^®^ (BO), Creos Xenogain^®^ (CX) and MinerOss^®^ X (MO) ± i-PRF were incubated with HOB. At day 3, 7, and 10, cell viability and metabolic activity as well as expression of ALP, OCN, and BMP-2, was examined.

**Results:**

For non-i-PRF groups, the highest values concerning viability were seen for CB at all time points. Pre-treatment with i-PRF increased viability in all groups with the highest values for CB-i-PRF after 3 and 7 and for CX-i-PRF after 10 days. For metabolic activity, the highest rate among non-i-PRF groups was seen for MO at day 3 and for CB at day 7 and 10. Here, i-PRF groups showed higher values than non-i-PRF groups (highest values: CB + i-PRF) at all time points. There was no difference in ALP-expression between groups. For OCN expression in non-i-PRF groups, CB showed the highest values after day 3, CX after day 7 and 10. Among i-PRF-groups, the highest values were seen for CX + i-PRF. At day 3, the highest BMP-2 expression was observed for CX. Here, for i-PRF groups, the highest increase was seen for CX + i-PRF at day 3. At day 7 and 10, there was no significant difference among groups.

**Conclusion:**

XBSM sintered under high temperature showed increased HOB viability and metabolic activity through the whole period when compared to XBSM manufactured at lower temperatures. Overall, the combination of XBSM with i-PRF improved all cellular parameters, ALP and BMP-2 expression at earlier stages as well as OCN expression at later stages.

## Introduction

The composition of bovine bone substitutes is similar to human bone due to the preserved microstructure of the osseous frame (Glowacki, 2005; Yamada and Egusa, 2018). These xenografts are known for osteoconduction and a low (if any) absorbability rate (Klein et al., 2013b; Dau et al., 2016). Deproteinization potentially allows elimination of transmission risk and antigenicity (Lei et al., 2015). However, different cleaning and manufacturing methods may affect the regeneration capacity of the a bovine bone substitute material. For example, manufacturing of deproteinized bovine bone by sintering consists of high temperature treatment with stepwise heating up to >1,000°C leading to the removal of all organic components including collagen (Lei et al., 2015). On contrary, manufacturing with lower temperatures usually comprise an additional chemical treatment, i.e., with sodium hydroxide with efficiently inactivated viruses (Tadic and Epple, 2004). Cerabone^®^ (Botiss, Zossen, Germany) is produced via three-stage temperature treatment including a final sintering at >1,200°C, hence all organic compounds are removed and potential prions, bacteria and viruses are eliminated. This preparation process might alter the microstructure (Perić Kačarević et al., 2018). However, it has been shown that Cerabone^®^ resembles the structure of natural bone with high porosity and rough surface (Trajkovski et al., 2018). Bio-Oss^®^ (Geistlich Pharma AG, Wolhusen, Switzerland) has a fiber-like surface with a much smaller crystal size (Barbeck et al., 2015; Perić Kačarević et al., 2018). It is manufactured at a lower temperature of 300°C followed by sodium hydroxide treatment (Kübler et al., 2004); thus, it is considered to be a hydroxyapatite ceramic with a high porosity including large interconnective pores and residual proteins (Kübler et al., 2004; Klein et al., 2009). Creos Xenogain^®^ (Nobel Biocare GmbH, Gothenburg, Sweden) is produced by sodium hypochloride treatment followed by heating under 400°C. MinerOss^®^ X (BioHorizons, Birmingham, United Kingdom) is also produced via low-heat processing of bovine bone, preserving the coarseness of bone with a high porosity.

In *in vitro* and *in vivo* studies, for injectable platelet-rich fibrin (i-PRF) a high share of leukocytes and platelets was proven. It promotes fibroblast migration and has a potential to release higher concentration of cytokines and selective growth factors over time when compared to PRP/L-PRF and A-PRF (Ghanaati et al., 2014; El Bagdadi et al., 2017; Miron et al., 2017; Wend et al., 2017; Choukroun and Ghanaati, 2018; Wang et al., 2018). Additionally, due to its consistency as well as composition, i-PRF can be used in combination with various biomaterials in order to increase their bioactivity in bone/soft tissue regeneration, and to improve healing in impaired wound healing cases (Wend et al., 2017; Miron and Zhang, 2018; Abd El Raouf et al., 2019). Thus, a combination of a bovine bone substitute material with i-PRF may be promising in terms of soft and hard tissue regeneration (Mourão et al., 2015). In our previous *in vitro* study (Kyyak et al., 2020), we compared an allograft and a bovine bone substitute material with and without i-PRF in regard of their effect on human osteoblasts’ viability, gap closure and metabolic activity. Here, the allogenic material showed an improved performance, possibly due to its (minimal) osteoinductive potential. The previous study was limited as there was only one commercially available bovine bone substitute material under examination. Thus, the aim of the study was to compare four different commercially available bovine bone substitutes with and without i-PRF for viability, metabolic activity, and differentiation of human osteoblasts. The null hypothesis was that pre-treatment of bovine bone substitute materials with i-PRF affects osteoblast viability, metabolic activity, and differentiation. A secondary hypothesis was that there are also differences between the different xenogenic materials.

## Materials and Methods

### Bone Substitute Materials

In the study, four bone substitute materials of bovine origin and their combination with i-PRF were included: cerabone^®^ (CB, botiss biomaterials GmbH, Zossen, Germany, granularity: 1–2 mm), Bio-Oss^®^ (BO, Geistlich Pharma AG, Wolhusen, Switzerland, granularity: 1–2 mm), CREOS Xenogain^®^ (CX, Nobel Biocare GmbH, Gothenburg, Sweden, granularity: 1–2 mm), MinerOss^®^ X (MO, BioHorizons, Birmingham, United Kingdom, granularity: 0.5–1 mm).

### I-PRF

In accordance with the ethical standards of the national research committee (Ärztekammer Rheinland-Pfalz, no. “2019-14705_1”), 10 ml peripheral venous blood per sample was collected from three healthy donors without severe illnesses after puncture of the cephalic or the median cubital vein. The vacutainer system and specific sterile plain vacuum tubes with additional silicone within their coating surface for solid (A-PRF+, Mectron, Carasco, Italy) and liquid PRF were used, respectively (iPRF, Mectron, Carasco, Italy). PRF was directly manufactured at a fixed angle rotor with a radius of 110 mm with 1,200 rpm and a relative centrifugal force of 177 g for 8 min (Duo centrifuge, Mectron, Carasco, Italy) after manufacturer’s instructions as described (Blatt et al., 2020).

### Cells

For the *in vitro* study, commercially available human osteoblasts from one donor were chosen (HOB, PromoCell, Heidelberg, Germany). A standard HOB medium was applied for cultivation including fetal calf serum (FCS, Gibco Invitrogen, Karlsruhe, Germany), Dulbecco’s modified Eagle’s medium (DMEM, Gibco Invitrogen), dexamethasone (100 nmol/l, Serva Bioproducts, Heidelberg, Germany), L-glutamine (Gibco Invitrogen), and streptomycin (100 mg/ml, Gibco Invitrogen). Cultivation of HOB was administered at the air temperature of 37°C, 95% humidity 95 and 5% of CO_2_. The passaging of HOB was carried out when reaching 70% confluence by application of 0.25% trypsin (Seromed Biochrom KG, Berlin, Germany). Passage five HOB were seeded in a density of 5 × 10^4^ cells per well. Afterward, 100 mg of each bone substitute material were added to the corresponding wells with HOB. Into the first half of the wells, 150 μl of i-PRF was applied, one well with each bone substitute material was left without i-PRF. Incubation of the compositions was divided into 3, 7, and 10 days at 37°C, 95% humidity, and 5% CO_2_. For negative controls, wells without bone substitute material as well as with i-PRF without bone substitute material were used.

### Cell Viability

For cell viability, CellTracker staining (Life Technologies, Thermo Fisher Scientific, Darmstadt, Germany; catalog number: C34552) was applied on day 3, 7, and 10. Red dye was produced following the manufacturer’s instructions. After the culture media was removed, Red dye was applied into wells and incubated for 30 min in 37°C. Serum-free medium was added, following the removal of the Red dye, and incubated for 30 min in 37°C. Red fluorescence was observed using a fluorescence BZ-9000 microscope (Keyence, Osaka, Japan). ImageJ software (ACTREC, Navi Mumbai, India) was used for cell quantification (Fuchs et al., 2009). At first, images (magnification 10×) were converted to grayscale. Through image subtraction, a correction of the background was conducted. Cell structures were extracted from the background using automatic thresholding and the area fraction (%) was calculated. Measures were conducted in triplication for each group and each time point (three time points).

### Cellular Metabolic Activity

Metabolic activity was measured by 3-(4,5-Dimethylthiazol-2-yl)-2,5-diphenyltetrazolium bromide (MTT) assay on day 3, 7, and 10. Briefly, MTT (200 μl, 2 mg/ml) was added to the wells and incubated for 4 h at 37°C. Culture medium was removed, and lysis buffer (10 ml) was pipetted into each well. Plates values were acquired using a fluorescence microplate reader (570 nm; Versamax, Molecular Devices, San Jose, CA, United States). Measures were conducted in triplication for each group and each time point (three time points).

### Expression of Bone Gene Markers

In accordance with Liu et al. (2003), the runx-2-dependent early osteogenic differentiation marker collagen alkaline phosphatase (ALP) as well as the late differentiation marker osteocalcin (OCN) was examined, whose expression is also highly controlled by runx-2 levels (Stein et al., 2004). ALP plays an important regulatory role during matrix mineralization (Hessle et al., 2002; Anderson et al., 2004). For osteogenic cells, the integrin subunits β_1_ and α_*v*_ have been shown to trigger effects of cytokines like bone morphogenetic protein 2 (BMP-2) (Lai and Cheng, 2005). OCN, which is only synthesized by mature osteoblasts, is directly associated with bone matrix mineralization as well (Lian et al., 1998, 2004; Klein et al., 2013a).

For this purpose, total RNA was extracted after day 3, 7, and 10 (Qiagen, Hilden, Germany). Subsequently, RNA was converted to cDNA with the help of iScript cDNA synthesis kit (BioRad, Hercules, United States) in accordance to manufacturers’ recommendations. For normalization, internal control Actin and GAPDH genes were used. The sequences of the primers are presented in Table 1. PCR was conducted using a CFX Connect Real-Time PCR Detection System (Bio-Rad, Germany) and SYBR Green Supermix (BioRad, Hercules, United States). Following proportions were applied: 11 μl of SYBR, 1 μl of primer sense, 1 μl of primer antisense, and 5 μl RNA-free water. The conditions of the thermal cycler were the following: first step—95°C for 3 min; second step (repeated 39 times)—95°C for 10 s, then 58°C for 30 s and finally 72°C for 20 s; final step—65°C for 0.5 s and then 95°C for 5 s. Quantification of gene expression was conducted through Ct value. Measures were conducted in triplication for each group and each time point (three time points).

**TABLE 1:** Primers Actin, GAPDH, ALP, OCN, and BMP-2 and their sequences.

Primers	Sequences
Actin	sense-GGAGCAATGATCTTGATCTT
	antisense-CTTCCTGGGCATGGAGTCCT
GAPDH	sense-AAAACCCTGCCAATTATGAT
	antisense-CAGTGAGGGTCTCTCTCTTC
ALP	sense- ACTGCAGACATTCTCAAAGC
	antisense-GAGTGAGTGAGTGAGCAAGG
OCN	sense-GSAAAGGTGCAGCCTTTGGT
	antisense-GGCTCCCAGCCATTGATACAG
BMP-2	sense-(1)-CCTGAAACAGAGACCCACCC
	antisense-(1)-TCTGGTCACGGGGAATTTCG

### Statistical Analyses

Data was converted into mean values with the estimate of its standard error of the mean (SEM) (for parametric data) and median values (for non-parametric data). Numbers were round off to second decimal place. Normal distribution was determined by Shapiro-Wilk test (SWT). For comparison of two groups, two-sided Student’s *t*-tests for paired samples (*t*-test) were applied in case of normal distributions. In case of non-normal distributions, Mann-Whitney test (MWT) was applied to compare two groups. Kruskal-Wallis rank sum test (KWT) was applied to compare all groups. A *p*-value of ≤0.05 was considered to be statistically descriptive significant. Data was visualized using bar charts with error bars.

## Results

### Cell Viability

**FIGURE 1:**
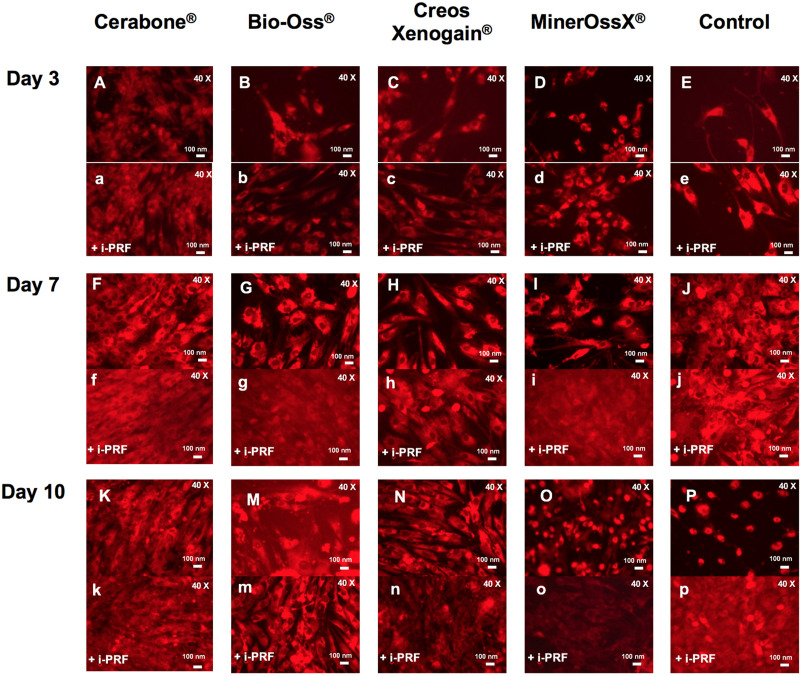
Representative figures of HOB viability visualized via red fluorescence (40×). BSM groups with **(a–k, m–p)** and without **(A–K, M–P)** i-PRF on day 3, 7, and 10.

**FIGURE 2:**
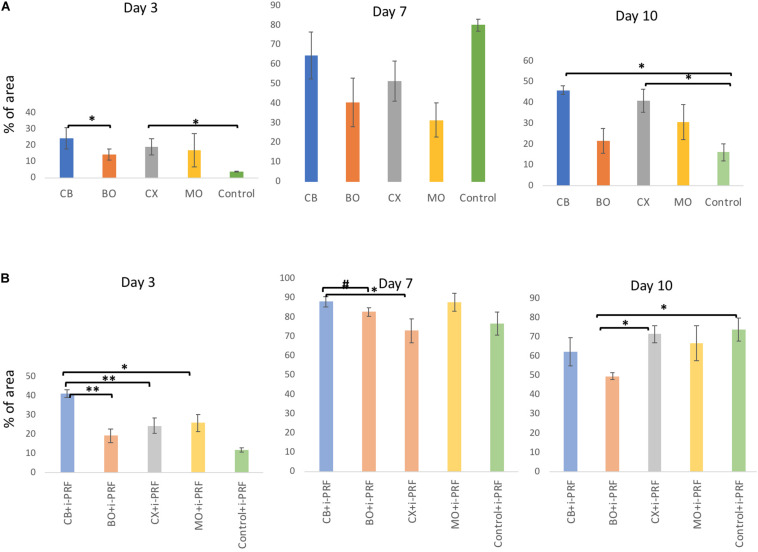
Bar charts: viable HOB (%) with XBSM **(A)** and XBSM with i-PRF **(B)** on day 3, 7, and 10 (area fraction, ImageJ). mean values; error bars show SEM; in triplication for each group and each time point, three time points. *p ≤ 0.05, *t*-test; **p ≤ 0.001, *t*-test; ^#^*p* ≤ 0.05, MWT.

At day 3, the highest cell viability was seen in the Cerabone^®^ (CB) group, which was significantly higher when compared to BioOss^®^ (BO; *p* ≤ 0.05, *t*-test) that showed the lowest indexes among all tested materials. Viability of Xenogain^®^ (CX) was significantly higher when compared to controls (*p* ≤ 0.05, *t*-test) (Figures 1, 2A). At day 7, controls had the highest cell viability (*p* > 0.05, MWT). Additionally, when compared to other bovine bone substitute material (XBSM) groups, CB presented the highest indexes (*p* > 0.05, *t*-test), followed by CX (*p* > 0.05, *t*-test) and MO (*p* > 0.05, *t*-test). At day 10, the highest viability value was seen for CB, followed by CX (when compared to controls (*p* ≤ 0.05, *t*-test), BO and MO (*p* > 0.05, MWT). The lowest rate showed BO (*p* > 0.05, MWT) (Figures 1, 2A and Table 2A).

**TABLE 2:** Cell viability of XBSM **(A)** and XBSM with i-PRF **(B)** by area fraction (%) on days 3, 7, and 10; experiments in triplication for each group and each time point, three time points.

A															

XBSM	Day3					Day 7					Day 10				
	CB	BO	CX	MO	Control	CB	BO	CX	MO	Control	CB	BO	CX	MO	Control
*Mean value with SEM/**Median	*24.27 + 6.63	*14.37 + 3.29	*19.11 + 4.81	*17.10 + 10.27	*3.75 ± 0.28	*64.46 + 11.99	**28.50	*51.51 + 10.21	**28.00	**80.53	*46.0 + 2.14	**24.77	*40.82 + 5.6	**30.42	*16.19 + 4.18

*Mean values for parametric data. **Median values for non-parametric data.															

**B**															

	**Day3**					**Day 7**					**Day 10**				
**XBSM**	**CB+i-PRF**	**BO+i-PRF**	**CX+i-PRF**	**MO+i-PRF**	**Control+i-PRF**	**CB+i-PRF**	**BO+i-PRF**	**CX+i-PRF**	**MO+i-PRF**	**Control+i-PRF**	**CB+i-PRF**	**BO+i-PRF**	**CX+i-PRF**	**MO+i-PRF**	**Control+i-PRF**

*Mean value with SEM/**Median	*41.25 + 1.92	*19.33 + 3.68	*24.49 ± 4.07	*25.95 + 4.51	**11.2	*87.98 + 2.44	**84.34	*73.08 + 6.11	**90.19	*76.7 + 5.86	**56.22	*49.71 + 1.86	*71.72 + 4.44	**64.42	*73.95 + 6.15

*Mean values for parametric data.

**Median values for non-parametric data.

At day 3, among i-PRF containing groups, significant higher values were observed for CB + i-PRF when compared to BO + i-PRF (*p* ≤ 0.001, *t*-test), CX + i-PRF (*p* ≤ 0.001, *t*-test), MO + i-PRF (*p* ≤ 0.05, *t*-test), and Control + i-PRF groups (*p* > 0.05, MWT). Control + i-PRF showed the lowest viability among i-PRF-groups (significant in comparison to all others, *p* > 0.05, MWT) and among i-PRF-XBSM groups, the lowest indexes were seen for BO + i-PRF (*p* > 0.05, *t*-test) (Figures 1, 2 and Table 2). At day 7, the highest increase in viability was observed in CB + i-PRF groups [when compared to BO + i-PRF (*p* ≤ 0.05, MWT), CX + i-PRF (*p* ≤ 0.05, *t*-test), controls (*p* > 0.05, *t*-test)] and MO + i-PRF groups (*p* > 0.05, MWT). At day 10, the highest viability was seen for controls, followed by CX + i-PRF [when compared to BO + i-PRF (*p* ≤ 0.05, *t*-test), CB + i-PRF, and MO + i-PRF (each: *p* > 0.05, MWT)] (Figures 1, 2B and Table 2B).

All i-PRF treated groups showed higher values in comparison to their equivalent pure non-i-PRF groups through the whole period. At day 7, values of all groups doubled in comparison to day 3 and were the highest of all the periods (Figures 1, 2 and Table 2).

### Cell Metabolic Activity

**FIGURE 3:**
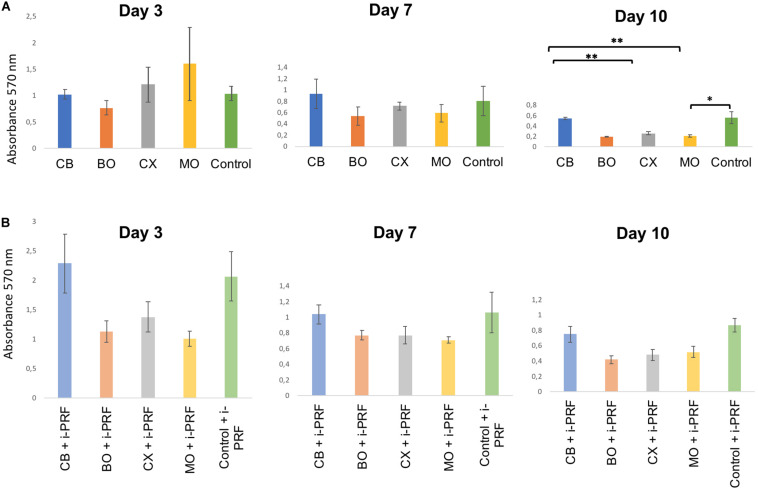
Bar charts: HOB metabolic activity (MTT) in groups with XBSM **(A)** and XBSM with i-PRF **(B)** on day 3, 7, and 10; mean values; error bars show SEM; in triplication for each group and each time point, three time points. *p ≤ 0.05, *t*-test; **p ≤ 0.001, *t*-test.

On the third day, MTT test showed the non-significant highest metabolic activity in MO (*p* > 0.05, *t*-test), followed by CX (*p* > 0.05, *t*-test). The least metabolic activity was observed for BO (*p* > 0.05, *t*-test). At day 7, the non-significant highest values were observed for CB, followed by controls (*p* > 0.05, *t*-test, MWT). At day 10, considerably higher values were seen in CB [when compared to BO (*p* > 0.05, MWT), CX (*p* ≤ 0.01, *t*-test), MO (*p* ≤ 0.05, *t*-test), and controls (*p* > 0.05, *t*-test)], followed by controls (in comparison to MO (*p* ≤ 0.05, *t*-test), CX (*p* > 0.05, *t*-test), and BO (*p* > 0.05, MWT) (Figure 3A and Table 3A). At day 3, 7, and 10 among i-PRF-groups, the non-significant highest metabolic activity was seen in CB + i-PRF (*p* > 0.05, *t*-test) and controls + i-PRF (*p* > 0.05, *t*-test). The lowest metabolic activity was seen for MO + i-PRF on day 3 and 7 as well as BO + i-PRF on day 10 (Figure 3B and Table 3B).

**TABLE 3:** HOB metabolic activity (MTT, absorbance 570 nm) in groups with XBSM **(A)** and XBSM with i-PRF **(B)** on days 3, 7, and 10; in triplication for each group and each time point, three time points.

A															

XBSM	Day 3					Day 7					Day 10				
	CB	BO	CX	MO	Control	CB	BO	CX	MO	Control	CB	BO	CX	MO	Control
*Mean value with EM/**Median	*1.02 ± 0.09	*0.77 ± 0.14	*1.21 ± 0.33	*1.6 ± 0.69	*1.04 ± 0.14	**1.19	*0.54 ± 0.16	*0.72 + 0.07	*0.59 ± 0.16	**0.76	*0.55 ± 0.02	**0.2	*0.26 ± 0.03	*0.21 ± 0.03	*0.56 ± 0.11

*Mean values for parametric data. **Median values for non-parametric data.															

**B**															

	**Day3**					**Day 7**					**Day 10**				
**XBSM**	**CB+i-PRF**	**BO+i-PRF**	**CX+i-PRF**	**MO+i-PRF**	**Control+i-PRF**	**CB+i-PRF**	**BO+i-PRF**	**CX+i-PRF**	**MO+i-PRF**	**Control+i-PRF**	**CB+i-PRF**	**BO+i-PRF**	**CX+i-PRF**	**MO+i-PRF**	**Control+i-PRF**

*Mean value with SEM/**Median	*2.29 ± 0.5	*1.13 ± 0.18	*1.38 + 0.26	*1.01 ± 0.14	*2.07 + 0.42	*1.04 ± 0.12	*0.77 ± 0.06	*0.77 ± 0.11	*0.71 ± 0.04	*1.06 ± 0.26	*0.75 + 0.1	*0.42 ± 0.05	*0.48 ± 0.07	*0.52 ± 0.07	*0.87 ± 0.09

*Mean values for parametric data.

**Median values for non-parametric data.

Overall, groups containing i-PRF showed a higher value than non-i-PRF groups, except for MO on day 3. Metabolic activity levels of all groups had a general tendency to decline through the whole period (Figure 3 and Table 3).

### Expression of Bone Gene Markers

#### Alkaline Phosphatase (ALP) Expression

On day 3, 7, and 10, all groups showed almost the same ALP expression level (*p* > 0.05, *t*-test), except MO, which didn’t show any expression at all through the whole period. On day 3 and 10, in the i-PRF-groups, the values were also almost on the same level (*p* > 0.05, *t*-test) with no expression in MO + i-PRF on day 10. At day 7, the highest expression was observed in BO + i-PRF [when compared to MO + i-PRF (*p* ≤ 0.05, *t*-test)], followed by CB + i-PRF (*p* > 0.05, *t*-test).

Overall, on day 3, 7, and 10, i-PRF-groups had slightly increased ALP expression over non-treated bone substitute materials, with the exception of CX + i-PRF on day 7 and MO + i-PRF on day 10.

#### Osteonectin (OCN) Expression

On day 3, CB had the highest OCN expression [significant in comparison to BO (*p* ≤ 0.05, *t*-test)]. On day 7, CX showed a significantly increased expression when compared to BO (*p* ≤ 0.05, *t*-test) and (non-significant) to CB (*p* > 0.05, *t*-test). On day 10, CX had significant highest values when compared to BO (*p* ≤ 0.05, *t*-test) and CB (*p* ≤ 0.001, *t*-test). There was no expression in MO through the whole period. On day 3, among i-PRF treated groups, the non-significant highest value of all groups was observed in CX + i-PRF (*p* > 0.05, *t*-test), the non-significant lowest in CB + i-PRF (*p* > 0.05, *t*-test). MO + i-PRF showed no OCN expression. On day 7, values of CX + i-PRF were higher than of MO + i-PRF (*p* ≤ 0.05, *t*-test) and CB + i-PRF (*p* > 0.05, *t*-test), followed by BO + i-PRF (*p* > 0.05, *t*-test). On day 10, CX + i-PRF showed the non-significant highest expression among treated groups (*p* > 0.05, *t*-test). Through the whole period, pre-treated groups showed increased expression rates when compared to non-treated XBSM, except for MO + i-PRF on day 3.

#### Bone Morphogenetic Protein 2 (BMP-2) Expression

Considering BMP-2 expression, on the day 3, the significant highest rate was observed in CX in comparison to BO (*p* ≤ 0.05, *t*-test), followed by CB (*p* > 0.05, *t*-test). On day 7 and 10, there was no considerable difference among the groups. Through the whole period, there was no expression in MO. On day 3, among the i-PRF-groups, CX + i-PRF demonstrated a significant increase when compared to MO + i-PRF (*p* ≤ 0.05, *t*-test) as well as a non-significant increase when compared to BO + i-PRF and CB + i-PRF (both *p* > 0.05, *t*-test). On day 7 and 10, there was no considerable difference among the groups with the exception of no expression of MO + i-PRF on day 10.

To sum up, on day 3, there was increased BMP-2 expression in all i-PRF-groups when compared to non-i-PRF-XBSM. On day 7, a positive effect of adding i-PRF on BMP-2 expression was seen in BO + i-PRF and MO + i-PRF, and on day 10 in BO + i-PRF.

## Discussion

For this *in vitro* study, four commercially available xenogenic, bovine bone substitute materials (XBSM)—alone and in combination with injectable platelet-rich fibrin (i-PRF) were evaluated regarding their biological effect on human osteoblast cells (HOB) after 3, 7 and 10 days. Cell viability, metabolic activity as well as expression of three bone regeneration markers (alkaline phosphatase (ALP), osteonectin (OCN), and bone morphogenic protein-2 (BMP-2) were analyzed. As a result, especially the high-sintered group showed beneficial *in vitro* effects when compared to low-sintered XBSM. Besides, addition of i-PRF to XBSM resulted in a significantly increased biological activity of HOB in most of the cases.

The main difference among the four XBSM is the preparation process, namely the temperature. They have a hydroxyapatite phase (Bohner, 2000), which causes a good biocompatibility due to similarity with crystalline phase of human bone, high porosity and micro-architecture (Laschke et al., 2007). Okumura et al. gave evidence that the reason of early osteogenesis on hydroxyapatite lies in the faster initial attachment of HOB (Stephan et al., 1999; Okumura et al., 2001). It was also reported that bovine hydroxyapatite materials treated at different temperatures show significant variation in osteoblastic activity because of changed surface roughness and biological performance (osteoconductivity) (Ong et al., 1998; Perić Kačarević et al., 2018; De Carvalho et al., 2019), which may result in different healing outcomes (Barbeck et al., 2015; Perić Kačarević et al., 2018). In our study, XBSM sintered at high temperatures [cerabone^®^ (CB)] showed a significantly increased HOB viability and metabolic activity when compared to other materials processed at lower temperatures. CB is composed of hydroxyapatite with traces of calcium oxide with a porous bone-like morphology (Tadic and Epple, 2004). Being sintered at a high temperature (>1,200°C), it loses all organic compounds. It was reported, that CB presents the highest level of hydrophilicity in comparison to Bio-Oss^®^ (BO) (Trajkovski et al., 2018). Besides, in 1,200°C sintered bovine hydroxyapatite, additional traces of NaCaPO4 and CaO were detected, which could result from decomposition of the bone carbonate and could improve HOB reaction (Tadic and Epple, 2004) as detected in the present study. Additionally, when considering the carbonate component, the influence of the surface energy of the bone substitute material may also increase initial HOB attachment and proliferation. Thus, the strengthening of the polar components of the dense surface of a bone substitute material may enhance HOB attachment and osteoconduction (Redey et al., 2000). The temperature of processing effects the elimination of carbonate content in the bone, which can be only initiated at 400°C and higher (Tadic and Epple, 2004). Besides, the high sintering temperature increases crystallinity, subsequently lowers biodegradation rates and increases volume stability (Ong et al., 1998; Bohner, 2000; Accorsi-Mendonça et al., 2008; Kusrini and Sontang, 2012; Riachi et al., 2012). On the other hand, it was stated in another study, that high-temperature sintering of a XBSM did not affect phase stability, densification behavior, fluid intrusion, and porosity when compared to non-sintered XBSM (Gehrke et al., 2019). In addition, no clinical long-term influence of osseous healing using differently processed bone substitute materials was found. Though, Kapogianni et al. (2019) analyzed samples from biopsies 6 months after sinus floor evaluation and after this considerable amount of time in a biological less demanding defect, no differences can be expected (Rickert et al., 2012; Kapogianni et al., 2019).

Despite the claim of no organic component, histological analyzes gave evidence for (xenogenic) organic remnants in XBSM treated under lower temperature, which may lead to decreased biocompatibility and osteoconductivity (Piattelli et al., 1999). BO is a carbonated hydroxyapatite, containing water, with porous granulate morphology and nanocristallinity (Tadic and Epple, 2004). It is manufactured at a temperature of 300°C, thus, is considered to include residual proteins (Kübler et al., 2004). In the present *in vitro* investigation, BO showed less distinct results for cell viability and metabolic activity as compared to other XBSMs. These findings are in accordance with other *in vitro* studies (Kübler et al., 2004; Liu et al., 2011). Sufficient osteogenic cell adhesion a bone substitute material is important for cellular proliferation, differentiation and matrix synthesis. Whereas initial cell attachment is based on unspecific cell-substrate interactions, later cell adhesion displays complex interactions between extracellular ligands and specific cellular receptors with high impact on further intracellular signal transduction (Keselowsky et al., 2007). Integrin receptors are transmembrane heterodimers consisting of non-covalently associated α and β sub-units. The sub-units β_1_ and α_*v*_ have affinity to extracellular matrix proteins like fibronectin, collagen, and osteonectin via the RGD tri-peptide sequence (Heller et al., 2018). Integrin-mediated outside-in-signaling has been shown to regulate osteogenic cytoskeleton organization and gene expression (Anselme, 2000; El-Ghannam et al., 2004). Furthermore, during osteoblast/substrate interactions, the expression of these adhesion molecules is modified according to distinct surface characteristics (Anselme, 2000; Klein et al., 2013a). In the present study, ALP, OCN (early as well as late osteogenic differentiation markers), and BMP-2 expression of BO without and with i-PRF was comparably high. In combination with i-PRF, Creos Xenogain^®^ (CX) showed a significantly elevated OCN expression through the whole period in comparison to other groups. However, the results of gene expression markers were inconsistent and it is possible that the inclusion of other gene expression markers such as type I collagen, Runt-related transcription factor 2 (Runx2) and Osteopontin would have shown different results.

Cell viability/metabolic activity of CX and MO more or less correlated to each other. MO is produced via low-heat processing of bovine bone, preserving the coarseness of bone with its high porosity. In a recent preclinical *in vivo* study, MO showed more osteogenic cells as well as more newly formed bone when compared to BO and autogenous bone (Esfahanizadeh et al., 2019). Nevertheless, the impact if cells are seeded on the well and the BSM is added afterward (Khanijou et al., 2020) or if the cells have been seeded on the BSM itself (Parisi et al., 2020) remains unclear.

Interactions of biomaterials such as BSM with the surrounding microenvironment define the respective biocompatibility and biochemical signaling pathways might play key roles in determining the materials’ success after implantation (Rahmati et al., 2020). *In vitro* assessment of cell metabolic activity may allow conclusions to be drawn about biocompatibility of biomaterials, and cells that are metabolically active are a precondition for osteoconductivity and osteoinductivity. But it should be clearly understood that *in vitro* studies still display only a limited part of the general *in vivo* set-up and there might be a substantial gap between cellular biocompatibility and *in vivo* models (Reichert et al., 2009; Rahmati et al., 2020). For example, surface characteristics of hydroxyapatite changes after getting in contact with blood proteins and extracellular matrix components (Herten et al., 2009). Thus, monotonous conditions of *in vitro* studies may distort *in vivo* results.

To the best of our knowledge, there are no *in vitro* studies on combination of i-PRF with different XBSMs. In a clinical study, Zhang et al. (2012) showed improvement in parameters of bone regeneration when adding PRF to XBSM but there was no statistical significance. In our previous *in vitro* study, we revealed that allogenic bone substitute material with i-PRF has a significant higher impact on HOB viability, migration and metabolic activity when compared to BO with i-PRF. Still, i-PRF-BO showed better results when compared to non-i-PRF-BO groups (Kyyak et al., 2020). In the present *in vitro* study, combination of i-PRF with xenogenic BSM significantly affected cell viability and metabolic activity of HOB, however not equally among the different XBSMs. Noteworthy is that material processed at high temperatures (CB + i-PRF) showed two times higher values of cell viability on day 3, when compared to other i-PRF-XBSM groups. Interestingly, all abovementioned indexes of i-PRF-CB were even higher than those of i-PRF-controls on day 3 and almost the same at most of the later time-points.

Still, it is not fully clear why the sintered XBSM showed better *in vitro* results above all studied materials—either with i-PRF or without. Therefore, further *in vitro* as well as preclinical studies for comparison between different bovine bone substitutes—for example using different amounts of bone substitute as they might have a dose-dependent effective range, using different media or using cells from different donors—are needed.

According to our *in vitro* study, it could be assumed that i-PRF addition to XBSM may have the potential to improve bone regeneration in clinical application. This might be of greatest importance, in particular, in cases with large complex defects or medically compromised patients. Additionally, XBSM sintered in a higher temperature showed an advantage over the XBSM treated in lower temperatures. The knowledge of the materials’ advantages leads to a better understanding of the regenerative processes and may improve the industrial production process.

## Conclusion

XBSM sintered under high temperature showed better HOB viability through the whole period as well metabolic activity on day 7 and 10 when compared to XBSM groups treated at lower temperatures. The same XBSM with addition of i-PRF showed even better HOB viability on day 3 and 7 as well as metabolic activity through the whole period in comparison to other XBSMs combined with i-PRF.

Overall, combination of XBSMs with i-PRF improves HOB viability and metabolic activity (except for one XBSM on day 3), ALP and BMP-2 expression at earlier stages as well as OCN expression at later stages *in vitro.*

## Data Availability Statement

The original contributions presented in the study are included in the article/supplementary material, further inquiries can be directed to the corresponding author/s.

## Author Contributions

PK, SK, and SB: hypothesis, concept, and methodology. SK, ES, DH, HS, DT, KS, SB, and PK: formal analysis and investigation. SK, BA-N, and PK: writing—original draft preparation. SK, ES, DH, HS, DT, KS, BA-N, SB, and PK: writing—review and editing. PK, KS, and BA-N: supervision. All authors contributed to the article and approved the submitted version.

## Conflict of Interest

The authors declare that the research was conducted in the absence of any commercial or financial relationships that could be construed as a potential conflict of interest.
